# Preparation of Interconnected Pickering Polymerized High Internal Phase Emulsions by Arrested Coalescence

**DOI:** 10.1021/acs.langmuir.2c01243

**Published:** 2022-08-26

**Authors:** Enes Durgut, Colin Sherborne, Betül Aldemir Dikici, Gwendolen C. Reilly, Frederik Claeyssens

**Affiliations:** † Kroto Research Institute, Department of Materials Science and Engineering, University of Sheffield, Sheffield S10 2TN, United Kingdom; ‡ Department of Materials Science and Engineering, INSIGNEO Institute for in Silico Medicine, The University of Sheffield, Sheffield S10 2TN, United Kingdom; § Department of Bioengineering, Izmir Institute of Technology, Urla, Izmir, 35433, Turkey

## Abstract

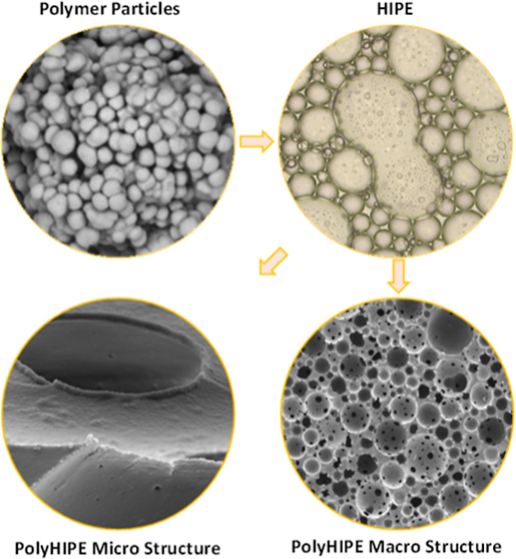

Emulsion templating is a method that enables the production of highly porous and interconnected polymer foams called polymerized high internal phase emulsions (PolyHIPEs). Since emulsions are inherently unstable systems, they can be stabilized either by surfactants or by particles (Pickering HIPEs). Surfactant-stabilized HIPEs form materials with an interconnected porous structure, while Pickering HIPEs typically form closed pore materials. In this study, we describe a system that uses submicrometer polymer particles to stabilize the emulsions. Polymers fabricated from these Pickering emulsions exhibit, unlike traditional Pickering emulsions, highly interconnected large pore structures, and we related these structures to arrested coalescence. We describe in detail the morphological properties of this system and their dependence on different production parameters. This production method might provide an interesting alternative to poly-surfactant-stabilized-HIPEs, in particular where the application necessitates large pore structures.

## 1 Introduction

Emulsion templating is a manufacturing method for creating porous interconnected polymeric materials. An emulsion is classified as a high internal phase emulsion (HIPE) when the internal droplet volume ratio is greater than 74% of the total volume fraction, which is the theoretical volume limit achievable from monodisperse spheres in a 3D close-packed face-centered cubic (FCC) array.^1^ The mixing of a crosslinkable hydrophobic monomer liquid with water creates a water-in-oil (w/o) emulsion, and polymerization of the oil phase (continuous phase) and removal of the water phase (internal phase) leaves behind a porous foam called a polymerized HIPE (PolyHIPE). The PolyHIPE’s internal structure replicates the emulsion at the monomer gel point. The porosity, average pore, and pore throat size are determined by tailoring various parameters such as the amount of the internal phase, surfactant, and stirring speed during the emulsification.^2^

Emulsions are thermodynamically unstable systems. When two immiscible liquids are mixed together without the stabilizing surfactant or particles, the droplet phase rapidly coalesces to minimize the contact area, and this causes the emulsion to separate back into its two bulk phases.^2^ Surfactants can be used to stabilize the emulsion by locating themselves at the interface between the two liquids to lower the interfacial tension and prevent droplet coalescence. Hypermer B246^3−8^ and Span 80^9,10^ are two common non-ionic surfactants used to stabilize w/o emulsions. Nevertheless, the surfactant removal from the final product is a laborious and costly process that can require intensive washing with solvents.^11^ Additionally, conventional PolyHIPEs possess small pores, typically 1–50 μm in size, which limits their application where the permeability is important^12^ or where large pores can be useful, for example, for vascularization in tissue engineering applications.^13^

In addition, particles can be used to stabilize the emulsion. These emulsions are termed Pickering emulsions. Here, particles with intermediate wettability localize at the oil/water interface. Silica oxide,^14^ titania,^15^ hydroxyapatite,^16^ and polystyrene^17^ are some of the commonly used particles required to prepare Pickering HIPEs. Particles to be used in HIPEs are generally subjected to a surface modification such as oleic acid^18^ or cetyltrimethylammonium bromide (CTAB)^19^ modification to tune their wettability. Rather than lowering the interfacial tension, particles form a solid barrier around the dispersed droplets, which inhibits the coalescence of emulsion droplets.^20^ The attachment/detachment energy of particles to/from the interface is higher compared to that of surfactants, which leads to superior emulsion stability in Pickering emulsions.^21^ Additionally, the incorporated particles may introduce further functionalities in the final PolyHIPE such as magnetic or light responsiveness^22^ or antibacterial properties.^23^

While particle stabilization offers several advantages over surfactant stabilization such as cost-effectiveness, higher stability, and lower toxicity (depending on the nature of the particles^24^) and potentially adds functionality,^22^ Pickering PolyHIPEs exhibit a closed pore structure. The formation of pore throats, which connect pores to each other, in poly-surfactant-stabilized-HIPEs is attributed to rupturing of the thin monomeric film between the neighboring droplets due to the polymerization-induced volume shrinkage^25^ or post-polymerization treatments.^26^ Alternatively, the pore throat formation due to the depletion attraction-induced droplet/pore coalescence has been proposed recently.^1^ According to the common view, the rigid particle shell around Pickering emulsion droplets increased the viscoelasticity of the continuous film separating two neighboring droplets, and the consequent thicker monomeric film resists rupturing during polymerization or post-polymerization treatments. The absence of interconnected pores prevents Pickering PolyHIPEs from being used for applications such as filtration or tissue engineering that require the use of substrates exhibiting an open cellular morphology. To overcome this problem, there have been several efforts to create interconnected Pickering PolyHIPEs. Inducing volume contraction during polymerization is achieved either by increasing the crosslinker content or by adding a co-monomer undergoing relatively higher shrinkage during polymerization.^27,28^ This approach requires the addition of substances that might not be relevant or applicable to the final application of the PolyHIPE or requires intensive crosslinking. Using a combination of a surfactant with the Pickering particles is a commonly used approach to obtain interconnected PolyHIPEs.^18,29,30^ However, while this system enables the fabrication of PolyHIPEs with large interconnected pores, the PolyHIPE will contain leachables, which might need to be removed. Alternatively, particle etching is demonstrated to obtain interconnected PolyHIPEs; however, this necessitates solvent extraction.^17^

We aimed to prepare 2-ethylhexyl acrylate–isobornyl acrylate–trimethylolpropane triacrylate (EHA/IBOA/TMPTA) Pickering PolyHIPEs, where the emulsion template was stabilized by polymeric microparticles (IBOA/TMPTA) sharing a similar chemical composition with the continuous phase of the HIPE. The HIPE was successfully synthesized, and the PolyHIPE is observed to exhibit an interconnected porous structure. The obtained PolyHIPE was compared with PolyHIPEs where the HIPE templates were stabilized by Hypermer B246 and hydrophobic silica (HDK H30) morphologically. Furthermore, the effects of the IBOA microparticle size and concentration and the internal phase fraction were also investigated morphologically. We hypothesized that the interconnected porous structure of Poly-IBOA-stabilized HIPE is due to partial but arrested coalescence of emulsion droplets, which is the phenomenon commonly observed in Pickering emulsions.

## 2 Materials

2-Ethylhexyl acrylate (EHA), isobornyl acrylate (IBOA), trimethylolpropane triacrylate (TMPTA), Tween 20, potassium persulfate (KPS), and 2-hydroxy-2-methylpropiophenone (photoinitiator, PI) were purchased from Sigma-Aldrich (Poole, UK). Hypermer B246-SO-M was received as a sample from Croda (Goole, UK). Pyrogenic silica (HDK H30) was purchased from Wacker.

## 3 Methods

### 3.1 Nomenclature of Samples

Synthesized IBOA microparticles are named according to the IB-X formula where IB stands for IBOA and X defines the size of particles: large(L, 724 nm), medium (M, 199 nm), and small (S, 103 nm). PolyHIPEs are defined using the abbreviation *U*(W)_*T*_ ,where *U* is the internal phase fraction, W is the stabilizer type, either Hypermer B246 (Hyp), IBOA (IB), ot silica (Si), and *T* is the stabilizer concentration. For example, 80(IB-L)_5_ defines the PolyHIPE having an 80% wt internal phase and stabilized by 5% wt of large IBOA particles. 80(Hyp)_5_ defines the PolyHIPE having an 80% wt internal phase and stabilized by 5% wt Hypermer B246.

### 3.2 Preparation of Microparticles

IBOA microparticles were prepared by ultrasound-assisted oil-in-water (o/w) emulsion polymerization, as listed in Table 1. Briefly, the continuous phase was prepared by dissolving respective amounts of Tween 20 in order to tune the emulsion droplet/particle size and potassium persulfate in 9 g of deionized water (dH_2_O) in a glass flask at room temperature. Next, 1 g of the internal phase consisting of the monomer/crosslinker blend was added to 9 g of the continuous phase in a glass flask. The ultrasonic processor horn was immersed approximately 2 cm deep into the mixture. The mixture was emulsified through sonication at 100 Watts, 30 kHz (Hielscher UP100H, Hielscher Ultrasound Technology) for a minute. The prepared emulsion was placed in a convection oven at 65 °C for 18 h for polymerization. Particles were washed with 30 mL of methanol for 15 min. The mixture was centrifuged at 14,000 RPM for 15 min, then the particle supernatant was removed. Particles were resuspended in 20 mL of water through sonication for a minute and dried at 65 °C overnight.

**Table 1:** IBOA and TMPTA Ratio (% wt), Average Particle Size (*D*_*p*_), and Polydispersity Index (PDI) of IBOA Microparticles

	internal phase		continuous phase			
ID	IBOA (%)	TMPTA (%)	Tween 20^a^ (%)	KPS^b^ (%)	*D*_*p*_ (nm)	PDI
IB-L	75	25	0.10	2	724	0.04
IB-M	75	25	0.50	2	198	0.03
IB-S	75	25	1.00	2	103	0.03

a Tween 20 concentration with respect to the continuous water phase (% wt).

b KPS concentration with respect to the internal organic phase (% wt).

### 3.3 Preparation of the EHA/IBOA PolyHIPE

The emulsion continuous phase was prepared by mixing a monomer blend consisting of EHA (63% wt), IBOA (21% wt), and the crosslinker TMPTA (16% wt). The respective amount of the stabilizer, either Hypermer B246, IBOA microparticles, or silica nanoparticles, and 0.1 g of PI were added and mixed into 4 g of the monomer blend (Table 2). Particles were dispersed in the monomer phase by sonication for a minute. The HIPE was prepared by the addition of respective amounts of dH_2_O dropwise using a syringe pump at 0.8 mL/min into the continuous phase while the mixture was being stirred using the overhead stirrer at 500 RPM (Pro40, SciQuip). The produced HIPE was poured onto a glass Petri dish and polymerized through the belt conveyor UV curing system (GEW Mini Laboratory, GEW Engineering UV). The polymers taken out of the dish were dried in an oven at 60 °C overnight. Additionally, a low internal phase emulsion (LIPE) with a 33% internal phase stabilized by the above-mentioned stabilizers was prepared while keeping the stabilizer to internal phase ratio the same as the HIPE’s.

**Table 2:** Density (*ρ*), Porosity (*P*_*Ø*_), Emulsion Droplet Size (*P*_*d*_), Polydispersity Index of the Emulsion Droplet Size (PDI *P*_*d*_), Pore Size (*P*_*p*_), Polydispersity Index of the Pore Size (PDI *P*_*p*_), Pore Throat Size (*P*_*t*_), Number of Pore Throats per Pore (#), and Degree of Openness (*D*_*o*_) of PolyHIPEs

sample	*ρ* (g/cm^3^)	*P*_Ø_	*P_d_* (μm)	PDI *P*_*d*_	*P*_*p*_ (μm)	PDI *P*_*p*_	*P*_t_ (μm)	#	*D*_o_
80(Hyp)_5_	1.02	78.29	10	0.25	8	0.61	6.14	16.40	0.083
80(IB-M)_5_	1.01	76.74	50	0.45	49	0.72	14.99	11.27	0.076
80(Si)_5_	0.55	57.70	80	0.28	85	0.84	N/A	N/A	N/A
80(IB-M)_1_	1.04	77.41	91	0.42	86	2.31	55.83	16.73	0.088
80(IB-M)_10_	0.98	75.26	34	0.42	21	0.93	7.49	4.46	0.026
80(IB-L)_5_	1.05	76.34	65	0.53	74	1.01	34.26	10.87	0.113
80(IB-S)_5_	1.00	75.82	39	0.35	27	0.89	11.37	3.66	0.035
85(IB-M)_5_	0.97	81.16	49	0.39	56	0.62	21.91	13.47	0.117
75(IB-M)_5_	0.96	72.43	49	0.80	54	0.81	12.49	4.73	0.033

### 3.4 Characterization

IBOA microparticles were 8 nm thick and gold-coated and were imaged using a scanning electron microscope (Inspect F, FEI) where the accelerating voltage and the spot size were 5 kV and 3, respectively. The oarticle size of IBOA microparticles was calculated by averaging the diameter of 300 particles measured from scanning electron microscopy (SEM) micrographs using the software ImageJ. The polydispersity index of particles was calculated according to the formula
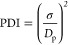
1where σ is the standard deviation and *D_p_* is the average particle size. Contact angle was measured by the sessile drop test and analyzed using the integrated software (FTA32, First Ten Angstroms) for IB-M and silica particles, which were placed on a double-sided tape and squeezed with the glass slide to smoothen the surface, as well as EHA/IBOA/TMPTA and IBOA/TMPTA polymer films photo-polymerized between two glass slides.

HIPEs and LIPEs were imaged under the light microscope (CX43, Olympus), and optical micrographs were captured using the integrated camera (DP27, Olympus). The average emulsion droplet size was calculated by averaging 100 emulsion droplets measured from optical micrographs using ImageJ. Viscosity of HIPEs was measured on a rheometer (AR2000, TA Instruments) by using a standard steel cone (40 mm 2°) at 25 °C.

The microarchitecture of PolyHIPE samples was investigated by SEM using a protocol similar to that used for IBOA microparticle imaging. The average pore size was calculated by averaging 250 pore sizes measured from SEM micrographs using ImageJ. The statistical correction factor was applied to reduce the error due to uneven sectioning according to the formula:^2,31^
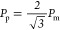
2where *P_p_* is the corrected average pore size and *P_m_* is the measured value.

The number of pore throats per pore and degree of openness are calculated from SEM micrographs as well; 15 highly interconnected pores, which are 2–2.5 times larger than the average pore size, were chosen; the number of pore throats on each chosen pore was counted and averaged to deduce the number of pore throats per pore. Additionally, the degree of openness was calculated according to the formula^2^
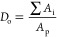
3where *D_o_* is the degree of openness, *A_i_* is the surface area of pore throats, and *A_p_* is the surface area of the pore. The pore surface is considered as a cap of a hemisphere.

The average pore throat size was measured using a mercury intrusion porosimeter (AutoPore V, Micrometrics), where the contact angle of mercury was 130° and the highest applied pressure was 30.000 psi. The bulk density of cylindrically cut PolyHIPE samples was calculated by dividing the measured mass by calculated volume from a known geometry. The skeletal density of PolyHIPEs were measured using a pycnometer (AccuPyc 1340, Micromeritics). The porosity of PolyHIPEs was calculated according to the formula
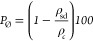
4where *P_Ø_* is the porosity, *ρ_sd_* is the skeletal density, and *ρ_c_* is the calculated density. The available particle surface is calculated according to the formula
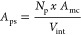
5where *A_ps_* is the available particle surface defining the sum of the mid-circular area of particles dispersed in the continuous phase per volume of the internal phase, *N_p_* is the number of particles in the continuous phase, *A_mc_* is the average mid-circular area of the given particle, and *V_int_* is the volume of the internal phase used to prepare the HIPE.

## 4 Results and Discussion

### 4.1 IBOA Microparticles

**Figure 1:**
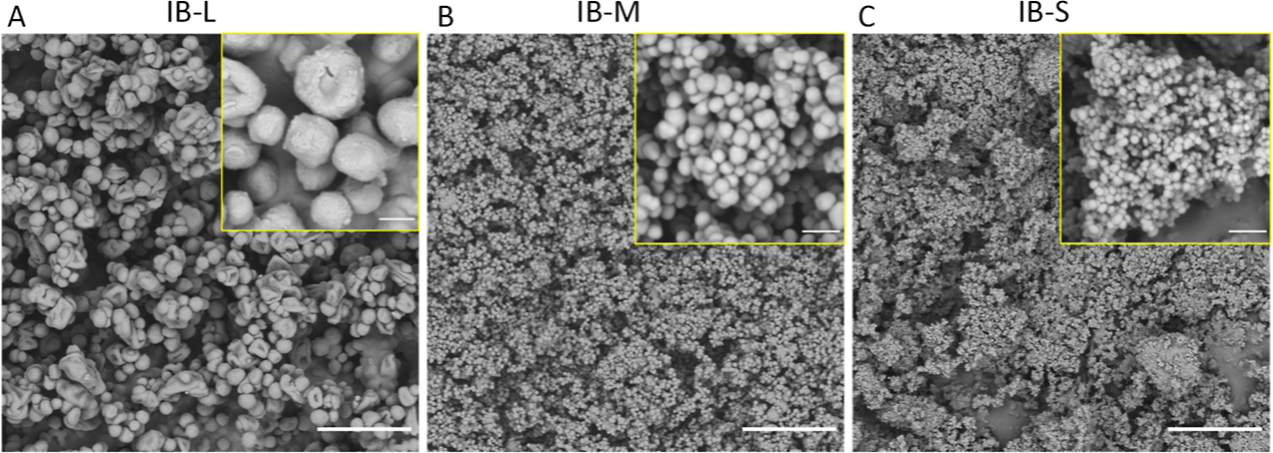
SEM images of IBOA microparticles; IB-L (A), IB-M (B), and IB-S (C). Scale bars are 5 μm and 500 nm for the main images and insets, respectively.

The particles listed in Table 1 were successfully synthesized by the emulsion polymerization method and are represented in Figure 1. Increasing emulsifier concentrations (Tween 20) yielded reduced average particle size, as previously reported.^32^ The diameters of the particles prepared using 0.1, 0.5, and 1% Tween 20 were measured to be 724, 198, and 103 nm, respectively. The polydispersity indices of all the produced particles were between 0.03 and 0.04. Irregular shaped particles were also observed in IB-L. The reduced amount of the stabilizer in the emulsion system might not have efficiently stabilized the emulsion droplets, leading to irregular shaped particles.

### 4.2 Emulsion Droplets

**Figure 2:**
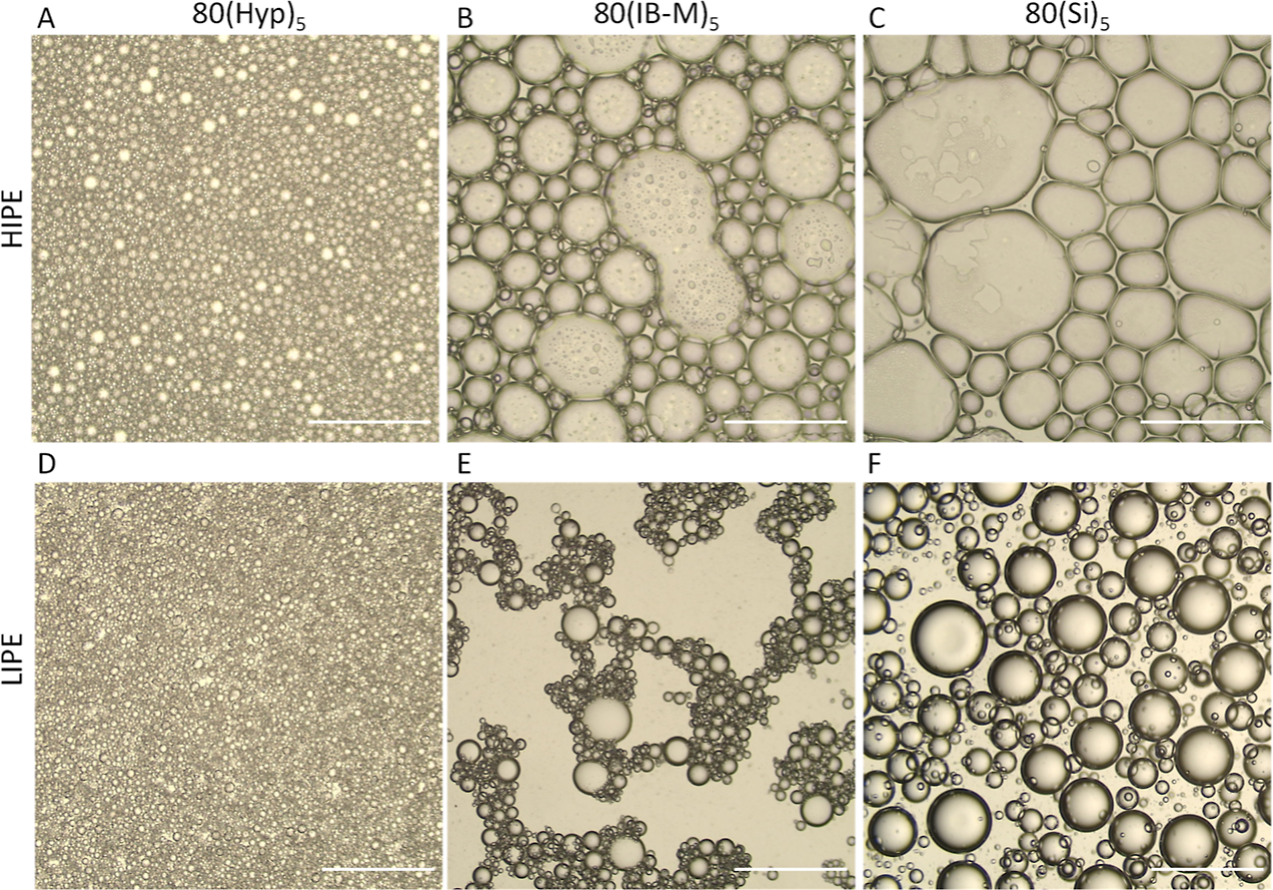
Optical micrographs of HIPEs (A–C) and LIPEs (D–F) stabilized by either Hypermer B246, IB-M, or silica nanoparticles. Scale bars are 200 μm.

80(Hyp)_5_, 80(IB-M)_5_, and 80(Si)_5_ HIPEs and LIPEs were successfully prepared. Microscopic evaluation of emulsion droplets was conducted, and the micrographs are presented in Figure 2. The average HIPE emulsion droplets sizes are 10.6, 50.7, and 80.1 μm for 80(Hyp)_5_, 80(IB-M)_5_, and 80(Si)_5_ HIPEs, respectively (Figure 2A–C). Emulsion droplets of Pickering HIPEs exhibit larger pores than that of surfactant-stabilized emulsion droplets, as reported previously.^33^ The 80(Si)_5_ HIPE exhibits a larger pore size than 80(IB-M)_5_, although the silica particles are smaller, 20 nm,^34^ than the synthesized IB-M. The silica-stabilized emulsion also exhibits a very high viscosity (see Figure S1), which increases with the amount of the internal phase (or water uptake). Indeed, the 80(Si)_5_ HIPE did not show any significant flow when its vial was turned upside down, in contrast with the two other emulsions. This high viscosity also means that high water incorporation is difficult to obtain due to inefficient mixing. Indeed, approximately 1.2 g (or 7.5%) of water was not incorporated in the 80(Si)_5_ HIPE. Thus, the high viscosity of the HIPE also has the effect of producing larger emulsion droplets and reduced maximum internal phase uptake because of inefficient mixing and the consequent reduced breakdown of large droplets into smaller ones.^20^

Partially coalesced droplets are observed in the 80(IB-M)_5_ HIPE (Figure 2B), as observed previously and attributed to arrested coalescence.^35,36^ Partial coalescence of multiple 80(IB-M)_5_ emulsion droplets is also observed and provided in Figure S2. To obtain the images of the HIPE, the emulsions were placed in between a glass slide and a coverslip, and this action might have induced coalescence in the HIPE and might not give a reliable overview on the 3D emulsion behavior. In order to image the organization of emulsion droplets, LIPEs were prepared while keeping the particle concentration to internal phase ratio the same as that for HIPEs. It was observed in optical micrographs of LIPEs (without placing them in between a microscope slide and a coverslip) that the IB-M-stabilized droplets form dense aggregates; larger droplets are covered with small droplets, and these small droplets seem to function as a bridging connection between the relatively larger droplets (Figure 2E). A similar droplet aggregation was observed previously and attributed to arrested coalescence.^37^ This behavior is distinct from that of both 80(Hyp)_5_ and 80(Si)_5_ HIPEs, and they do not exhibit the level of droplet aggregation shown in the 80(IB-M)_5_ LIPE.

The difference in droplet aggregation between 80(IB-M)_5_ and 80(Si)_5_ might be associated with the particle localization at the oil/water interface. However, the contact angles of IB-M and silica nanoparticles are very similar with values of 125.6 and 126.1°, respectively. Given that our measurements are a convolution of hydrophobicity and surface roughness, as induced by the nanoparticles, the inherent hydrophilicity/hydrophobicity of the materials tends to increase when cast on a surface in the nanoparticle form, as highlighted in detail in ref (38). We measured the contact angle on cast films of EHA/IBOA/TMPTA (the same composition as that of the continuous phase) and IBOA/TMPTA (the same composition as that of IB-M) to be 63.9°. Therefore, the estimation of particle localization at the oil/water interface is difficult in the current experimental design.

Total stability of 80(Si)_5_ emulsion droplets might be due to the prevention of emulsion droplet contact either by total coverage of emulsion droplets by silica functioning as a mechanical barrier or resistance of the viscoelastic thin monomer film between emulsion droplets. For 80(IB-M)_5_ emulsion droplets, the insufficient coverage of droplets with particles can lead to initiation of coalescence but being arrested due to migration of the particles to the contact point (or the necking region) and jamming to prevent the interfacial mobility.^39^ On the other hand, interparticle attraction forces might be another mechanism resulting in flocculated emulsion droplets. In either case, it is expected to obtain an interconnected porous structure upon polymerization of the 80(IB-M)_5_ template due to their flocculated state. The pore throat formation might be due to thin film rupture between neighboring pores or partial but arrested coalescence of emulsion droplets. If the pore throat formation is due to the arrested coalescence of droplets, it is expected that observe dense particle layer surrounding pore throats and in-between pores is observed due to particle jamming at the necking region of these partially coalesced emulsion droplets.

### 4.3 Effect of the Stabilizer Type on the PolyHIPE Morphology

**Figure 3:**
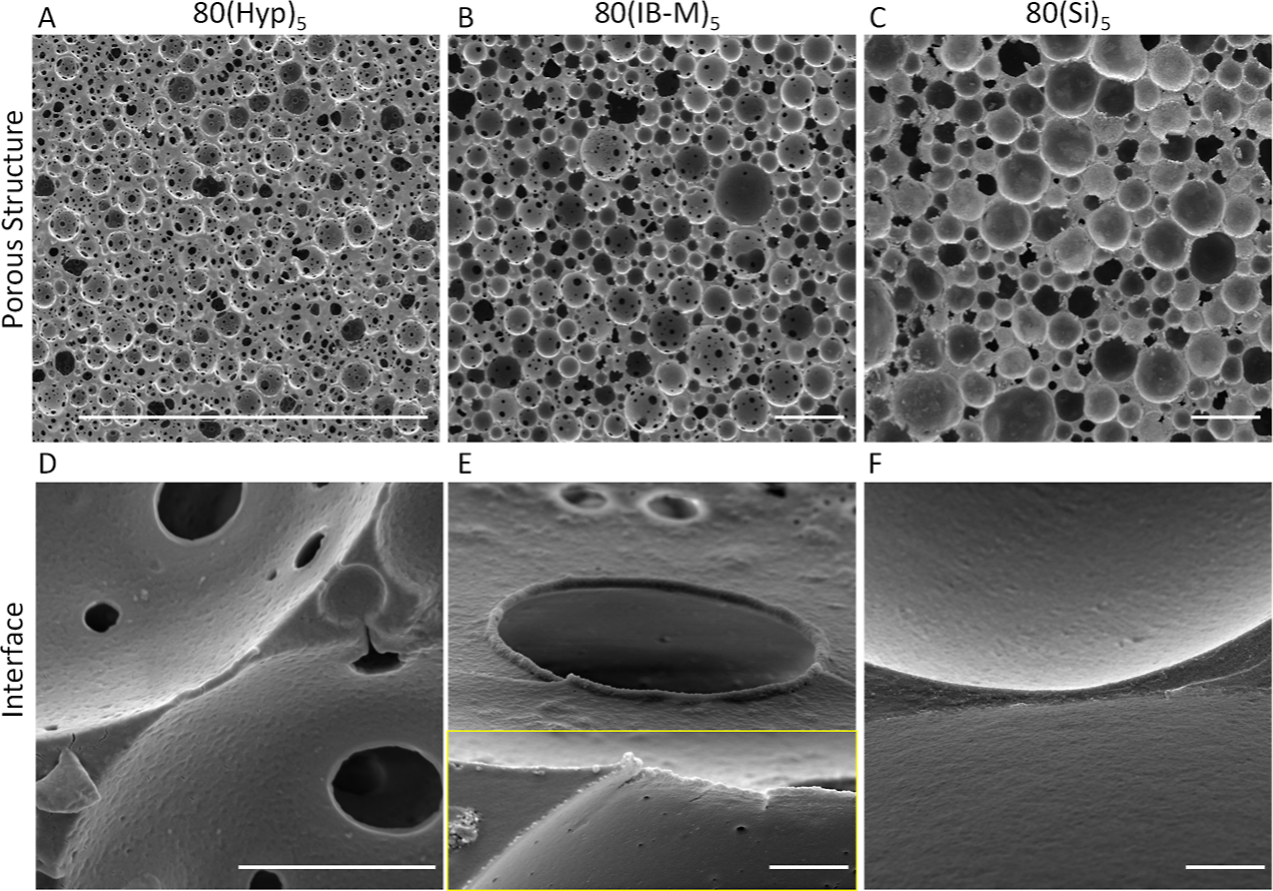
SEM images 80(Hyp)_5_, 80(IB-M)_5_, and 80(Si)_5_, focusing on the porous structure (A–C) and interface (D–E). Images from the same region, one focusing on the pore throat and the other focusing on the interface, are merged (*E*). Scale bars are 250 μm (A–C) and 5 μm.

PolyHIPEs listed in Table 2 were successfully synthesized and morphologically investigated through the acquired SEM images provided in Figure 3. The pore sizes of PolyHIPEs are 8, 49, and 85 μm for 80(Hyp)_5_, 80(IB-M)_5_, and 80(Si)_5_, in correlation with the emulsion droplet size observed under the light microscope.

Interestingly, 80(IB-M)_5_ exhibits an interconnected porous structure, which is an uncommon morphology for Pickering PolyHIPEs.^20^ However, pore throats of 80(IB-M)_5_ differ from pore throats of 80(Hyp)_5_ in two ways. First, 80(Hyp)_5_ represents a nearly homogeneous distribution of pore throats, regardless of the pore size. On the other hand, 80(IB-M)_5_ exhibits an interconnected porous structure especially on the relatively larger pores together with submicron-sized pore throats. Second, relatively smaller pores of 80(IB-M)_5_ are generally closed; however, they contain submicron pore throats (Figure 3E). Additionally, pore throats observed in 80(IB-M)_5_ are encircled with a dense particle layer. This observation is considered as an indication of pore throat formation due to partial but arrested coalescence of emulsion droplets. While 80(Si)_5_ does not exhibit pore throats, thinned pore walls are occasionally observed. Thinned regions of the pore walls are considered susceptible regions for pore throat formation during post-processing and commonly observed in Pickering PolyHIPEs.^33,40^

Furthermore, a thin polymer film separating two neighboring pores is observed in 80(Hyp)_5_ (Figure 3D) and 80(Si)_5_ (Figure 3F) but is absent between the highly interconnected pores of 80(IB-M)_5_ (Figure 3E). Instead, there is a curved pore–pore junction, which is delineated by a dense particle layer. The similarity between pore throats and the pore–pore junction leads us to conclude that any pore throat in the SEM results in a pore–pore junction (which is a throat in the transverse view). Additionally, micron-sized pores are observed in close proximity to the larger pores. These micron-sized pores correlate to the bridging emulsion droplets observed in LIPEs. Therefore, it is concluded that the conventional pore throats are due to partial coalescence of emulsion droplets, whose further coalescence was arrested by the dense particles jamming the necking region of droplets, and submicron openings on the pore surface are due to partial coalescence of micron-sized droplets.

### 4.4 IBOA Microparticle-Stabilized PolyHIPEs

Assuming that the interconnected porous structure observed in 80(IB-M)_5_ is due to the partial and arrested coalescence of emulsion droplets, it is expected to observe increased interconnectivity as the available particle surface to stabilize the internal phase decreases; the more particle-free regions on emulsion droplets would be available for droplets to contact. The available particle surface is defined as the particle mid-circular area in a given weight fraction per volume of the internal phase. Therefore, 80(IB-M)_5_ is chosen as a control, and the available particle surface is reduced by decreasing the particle concentration [80(IB-M)_1_, 0.19 cm^–1^], increasing the particle size [80(IB-L)_5_, 0.27 cm^–1^], and increasing the internal phase fraction [85(IB-M)_5_, 0.66 cm^–1^]. Samples were compared with their higher available particle surface counterparts; 80(IB-M)_10_ (1.88 cm^–1^), 80(IB-S)_5_ (1.88 cm^–1^), and 75(IB-M)_5_ (1.25 cm^–1^).

**Figure 4:**
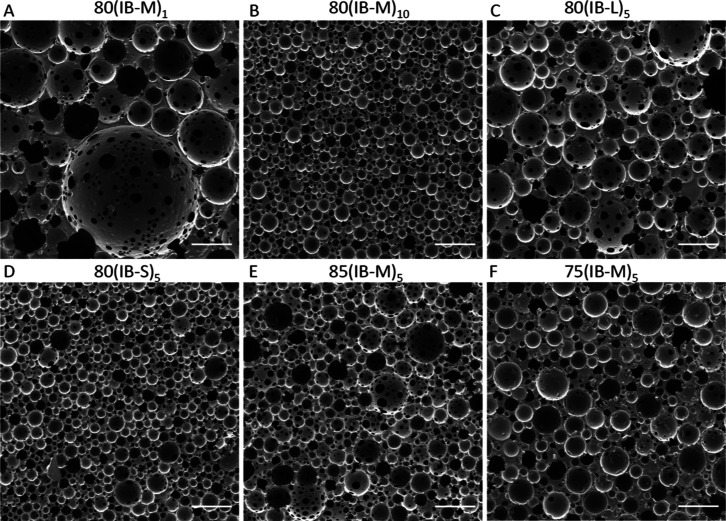
SEM images of PolyHIPEs stabilized by IBOA microparticles demonstrating the overall porous structure. Scale bars are 250 μm.

SEM images demonstrating the porous structure of prepared PolyHIPEs are represented in Figure 4. The average pore size of PolyHIPEs increases as the particle concentration reduces (Figure 4A,B)^41^ or particle size increases (Figure 4C,D),^42^ in accordance with the previous reports. The average pore throat size, number of pore throats per pore, and degree of openness are increased in samples with low particle availability.

Interestingly, the internal phase fraction did not significantly affect the pore size when the internal phase fraction was increased from 75 to 85% (Figure 4E,F). This result contradicts the previous reports, where the increase in the internal phase fraction leads to an increased pore size due to the limited coalescence phenomenon; the complete coalescence of emulsion droplets is required to reach the total surface coverage.^43,44^

There can be two arguments to explain the unaffected pore size as the internal phase fraction is increased: (1) there might be a sufficient number of particles to stabilize the increased internal phase fraction. In this case, the reduction in pore size as the particle concentration is increased [80(IB-M)_5_ vs 80(IB-M)_10_] would not be observed. However, as shown in Table 2, an increase in particle concentration from 5% to 10% reduces the average pore size. Alternatively, (2) the HIPE might not take up the increased internal phase fraction. In this case, there should not be a porosity difference between 75(IB-M)_5_ and 85(IB-M)_5_. However, the porosity increased from 72.43 to 81.16 as the internal phase fraction is increased from 75 to 85%. Additionally, according to mercury intrusion measurements, the total pore surface areas are 26, 27, and 26 m^2^/g, while the total pore volumes are 2.88, 3.61, and 4.98 mL/g for the internal phase fractions 75, 80, and 85%, respectively. On the other hand, the interconnectivity is increased, as deduced from the increase in the average pore throat size, number of pore throats per pore, and degree of openness as the internal phase fraction is increased from 75 to 85% (Table 2). This finding is considered as another supporting fact for the formation of interconnected porous structures due to arrested coalescence. Since the weight percentage of particles used are the same, they can stabilize the same amount of interfacial area. Insufficiency of particles as the internal phase fraction is increased allows a higher number of emulsion droplets to partially coalesce. Since the interfacial area of partially coalesced droplets is lower than two separate droplets, the interfacial area is balanced due to partial coalescence without significantly affecting the pore size but increasing interconnectivity.

**Figure 5:**
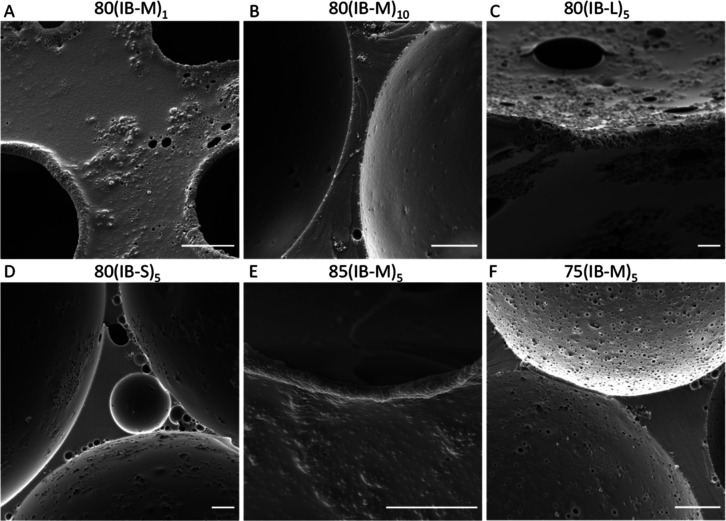
SEM images of PolyHIPEs stabilized by IBOA microparticles focusing on the pore surface and interfaces. Scale bars are 5 μm.

SEM images focusing on the microarchitecture of PolyHIPEs are provided in Figure 5. Similar to 80(IB-M)_5_, particle-covered pore throats (Figure 5A, E), pore–pore junctions similar to pore throats (Figure 5C), micron-sized pores (Figure 5B–D,F), and submicron pore throats are observed. The continuous polymer film separating two pores is occasionally observed in 80(IB-M)_10_ and 80(IB-S)_5_ (Figure 5B,D), in correlation with the reduced interconnectivity compared to that of other IB PolyHIPEs.

**Figure 6:**
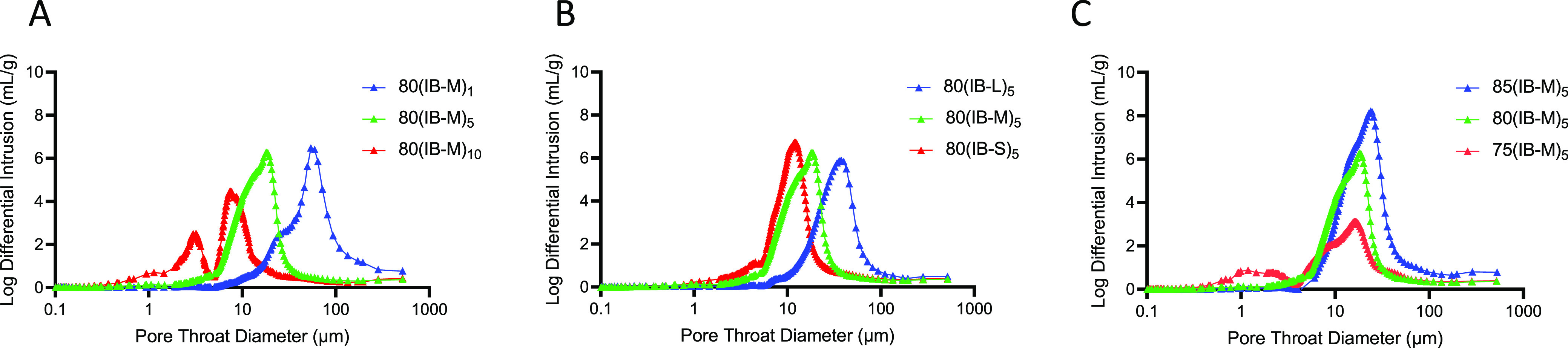
Pore throat diameter (μm) as a function of log differential intrusion (mL/g) obtained from a mercury intrusion porosimeter for the samples where the particle concentration (A), particle size (B), and internal phase fraction (C) are tuned.

Micron-sized pores are observed in all samples, but their frequency varies. However, it is hard to evaluate them quantitatively. While the formation of submicron pore throats is also attributed to partial coalescence of micron-sized pores, particle leaching from the pore surface might be another mechanism or a co-mechanism to induce their formation. Mercury intrusion was used to evaluate if submicron pore throats connected pores to each other. It has previously been reported that mercury intrusion provides a pore throat distribution rather than a pore size in PolyHIPEs.^31^ The presence of submicron pore throats can be seen in Figure 6. A bimodal pore throat size distribution is observed in high particle availability samples; 80(IB-M)_10_, 80(IB-S)_5_, and 75(IB-M)_5_.

Alternatively, the effect of leftover or adsorbed Tween 20 to the particle surface on the PolyHIPE morphology is also considered. Tween 20 is a surfactant with a high hydrophilic–lyophilic balance (16.7), which preferentially stabilizes oil-in-water emulsions. Indeed, Tween20 does not stabilize EHA/IBOA PolyHIPEs and any high-water-ratio emulsions rapidly experience phase inversion. The production of polymer spheroids due to subsequent polymerization of double emulsions (oil-in-water type within the water droplets) has been reported previously.^33,44^ Thus, as these artifacts were not observed, any effects of potential leftover Tween 20 after washing can be discarded. On the other hand, Tween 20 might be adsorbed onto the particle surface and affect their wettability and associated localization. Attenuated total reflection (ATR) was conducted on particles, bulk polymer, and Tween 20, but none of the peaks associated with Tween 20 were observed in washed particles (results not shown).

**Figure 7:**
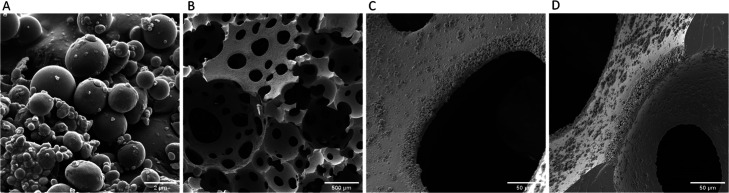
SEM images of IBOA particles prepared through photopolymerization without a surfactant (A) and PolyHIPEs synthesized when the emulsifier-free particles were used as a sole stabilizer: overall porous structure (B), pore throat (C), and pore interface (D).

In order to evaluate if the Tween 20 adsorbs on the particles and affects the PolyHIPE morphology, emulsifier-free particles were synthesized. This was performed by immediate photopolymerization after emulsification via sonication since the oil droplets have a tendency to coalesce in the absence of a surfactant. These particles were used to prepare PolyHIPEs using the same recipe as that used to prepare the poly-IBOA-stabilized HIPEs. Similar morphological features were observed, as previously discussed, such as pore throats (Figure 7B), particle layers surrounding the pore throats (Figure 7C), and no thin polymer film separating the pores but instead a dense layer of particles (Figure 7D), indicating that the effect of any absorbed Tween20 is minimal on the final PolyHIPE morphology. On the other hand, the submicron pore throats or pore throats at the scale of the stabilizing particles are not observed. This observation eliminates the possibility of their formation due to particle leaching.

**Figure 8:**
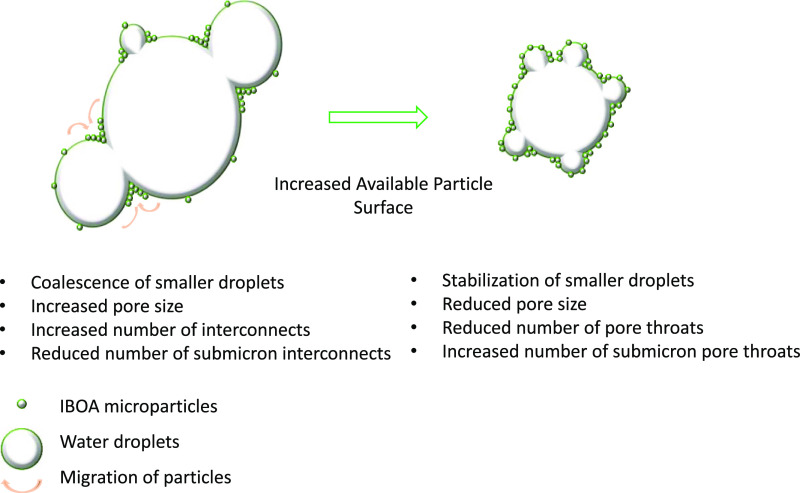
Schematic demonstration of the proposed pore throat formation due to arrested coalescence.

While the particle size, particle concentration, and internal phase fraction affect the HIPE in a complex manner, the results obtained in these experiments can be simplified and demonstrated in Figure 8; when the emulsion is particle-insufficient, the frequency of droplet contact is increased. Particles migrate to the necking region between droplets to arrest further the coalescence of droplets. Due to the scarcity of particles, micron-sized droplets cannot be stabilized; thus, they coalesce. Consequently, PolyHIPEs with a larger pore size, a high number of interconnects, a reduced number of micron-sized pores, and associated submicron interconnects are produced (0.19–0.66 cm^–1^). An increase in particle availability first manifests itself as a loss of interconnects due to reduced particle free regions on emulsion droplets rather than affecting the emulsion/pore size, as observed upon decreasing the internal phase fraction from 85 to 75% (0.66–1.25 cm^–1^). A further increase in the available particle surface leads to efficient stabilization of smaller droplets as well as micron-sized droplets. Efficient coverage of emulsion droplets prevents their partial coalescence; however, particle-covered-micron sized droplets either function as a stabilizer or their partial coalescence leads to the induction of submicron pore throats (1.25–1.88 cm^–1^).

## 5 Conclusions

In this study, the formation of interconnected porous Pickering PolyHIPEs with a bimodal pore throat size distribution is demonstrated without any surfactant and/or particle surface modification. The interconnected porous structure is attributed to arrested coalescence and supported by morphologic similarities between pore throats and the pore–pore junctions, where both are covered by a dense particle layer. To the best of our knowledge, this is the first time that pore throat formation due to arrested coalescence in PolyHIPEs has been demonstrated. Such PolyHIPEs can be used when the purity of the material is important since the stabilizer has the same composition as that of the material itself. Additionally, due to tunable openness and the larger pore size, as compared to that of poly-surfactant-stabilized-HIPEs, these structures will likely find interesting new applications as tissue engineering scaffolds. Additionally, the existence of a bimodal distribution of pore throats (micron and sub-micron) might have interesting consequences for mass transport in these porous materials and might lead to new filtering devices, insulation materials, or absorbent foams for environmental applications. On the other hand, the effect of interparticle and monomer–particle interactions, the forces during the polymerization such as volume shrinkage or depletion attraction on partial coalescence of pores, and the applicability of this method to other systems have not been elucidated yet.

## Supporting Information Available

Viscosity of HIPEs [80(Hyp)_5_, 80(IB-M)_5_, and 80(Si)_5_]; optical micrographs of the 80(IB-M)_5_ HIPE, demonstrating the droplet coalescence; and optical micrographs of IBOA-stabilized HIPEs (PDF)
