# Supplementary material for: Preparation of Interconnected Pickering Polymerized High Internal Phase Emulsions by Arrested Coalescence
Source: Langmuir. 2022 Aug 26;38(36):10953–62. doi: 10.1021/acs.langmuir.2c01243 (PMC9476866; doi:10.1021/acs.langmuir.2c01243)
Supplement: Supplementary file 1 — la2c01243_si_001.pdf [file la2c01243_si_001.pdf]

Supporting Information

Preparation of Interconnected Pickering Polymerised High Internal Phase Emulsions by Arrested Coalescence

*Enes Durgut [a, b], Colin Sherborne [a], Betül Aldemir Dikici [c], Gwendolen C. Reilly [a, b], Frederik Claeyssens [a, b, *]*

[a] *Kroto Research Institute, Department of Materials Science and Engineering, University of Sheffield, Sheffield, United Kingdom*

[b] *Department of Materials Science and Engineering, INSIGNEO Institute for In Silico Medicine, The University of Sheffield, Sheffield, United Kingdom*

[c] *Department of Bioengineering, Izmir Institute of Technology, Urla, Izmir, 35433, Turkey*

***Corresponding Author:** f.claeyssens@sheffield.ac.uk

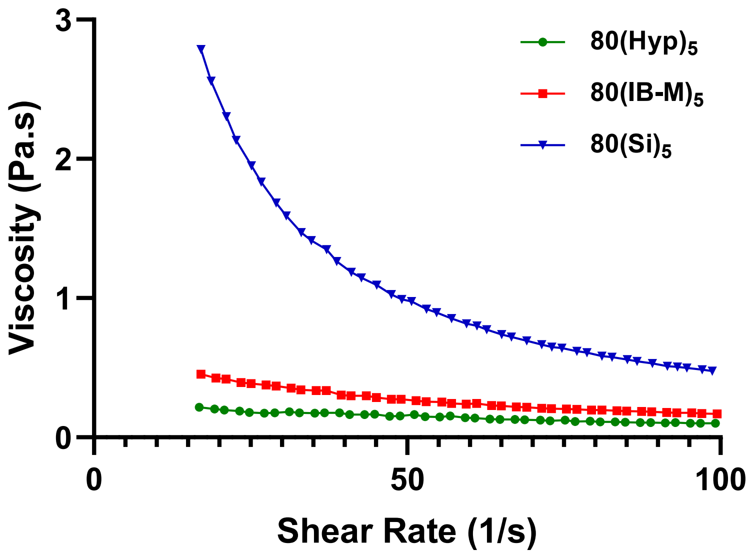

Figure S1: The viscosity of HIPE samples as a function of shear rate.

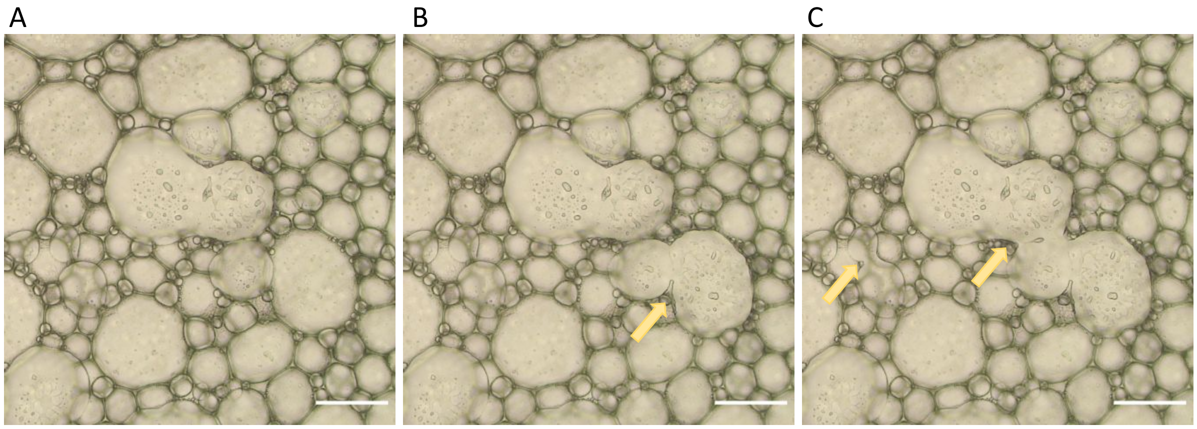

Figure S2: Demonstration of partial coalescence of emulsion droplets in 80(IB-M)$_5$.The necking of emulsion droplets is indicated by orange arrows. Images were captured within a minute. Scale bars are 100 µm

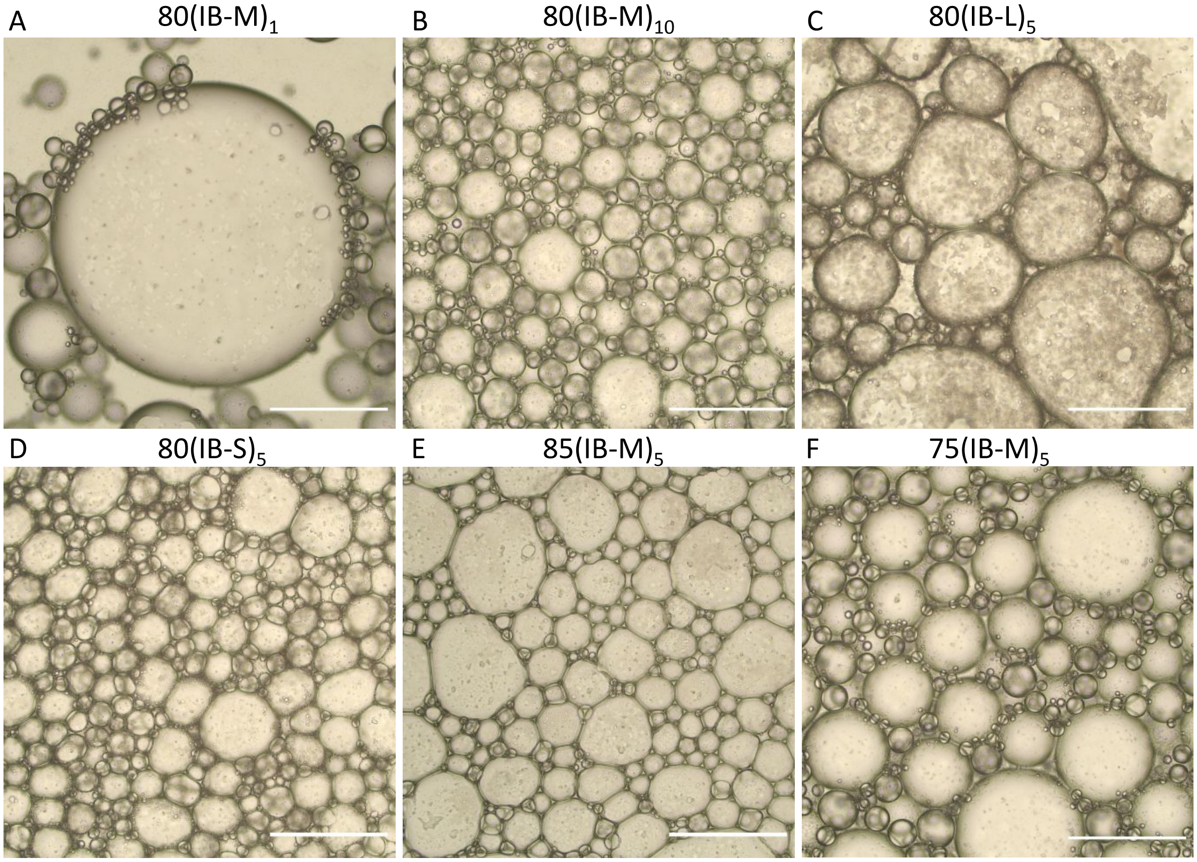

Figure S3: Optical micrographs of HIPEs stabilized by IBOA particles. Scale bars are 200 µm.
